# *Schmidtea mediterranea *phylogeography: an old species surviving on a few Mediterranean islands?

**DOI:** 10.1186/1471-2148-11-274

**Published:** 2011-09-26

**Authors:** Eva M Lázaro, Abdul Halim Harrath, Giacinta A Stocchino, Maria Pala, Jaume Baguñà, Marta Riutort

**Affiliations:** 1 Institut de Recerca de la Biodiversitat and Dept. Genètica, Facultat de Biologia, Universitat de Barcelona, Av Diagonal, 645, Barcelona 08028, Spain; 2 Zoology Department, College of Science, King Saud University, P.O. Box 2455, Riyadh 11451, Saudi Arabia; 3 Laboratoire de Biologie de la Reproduction et du Développement Animal, Département de Biologie, Faculté des Sciences de Tunis, Université de Tunis El-Manar, Campus Universitaire, 2092 El Manar, Tunis, Tunisia; 4 Dipartimento di Zoologia e Genetica Evoluzionistica, dell'Università di Sassari, Corso Margherita di Savoia 15, 07100 Sassari, Italy

## Abstract

**Background:**

*Schmidtea mediterranea *(Platyhelminthes, Tricladida, Continenticola) is found in scattered localities on a few islands and in coastal areas of the western Mediterranean. Although *S. mediterranea *is the object of many regeneration studies, little is known about its evolutionary history. Its present distribution has been proposed to stem from the fragmentation and migration of the Corsica-Sardinia microplate during the formation of the western Mediterranean basin, which implies an ancient origin for the species. To test this hypothesis, we obtained a large number of samples from across its distribution area. Using known and new molecular markers and, for the first time in planarians, a molecular clock, we analysed the genetic variability and demographic parameters within the species and between its sexual and asexual populations to estimate when they diverged.

**Results:**

A total of 2 kb from three markers (*COI*, *CYB *and a nuclear intron *N13*) was amplified from ~200 specimens. Molecular data clustered the studied populations into three groups that correspond to the west, central and southeastern geographical locations of the current distribution of *S. mediterranea*. Mitochondrial genes show low haplotype and nucleotide diversity within populations but demonstrate higher values when all individuals are considered. The nuclear marker shows higher values of genetic diversity than the mitochondrial genes at the population level, but asexual populations present lower variability than the sexual ones. Neutrality tests are significant for some populations. Phylogenetic and dating analyses show the three groups to be monophyletic, with the west group being the basal group. The time when the diversification of the species occurred is between ~20 and ~4 mya, although the asexual nature of the western populations could have affected the dating analyses.

**Conclusions:**

*S. mediterranea *is an old species that is sparsely distributed in a harsh habitat, which is probably the consequence of the migration of the Corsica-Sardinia block. This species probably adapted to temperate climates in the middle of a changing Mediterranean climate that eventually became dry and hot. These data also suggest that in the mainland localities of Europe and Africa, sexual individuals of *S. mediterranea *are being replaced by asexual individuals that are either conspecific or are from other species that are better adapted to the Mediterranean climate.

## Background

*Schmidtea mediterranea *Benazzi et al., 1975 is a freshwater planarian (order Tricladida, suborder Continenticola, phylum Platyhelminthes) that is mostly known to the scientific community for its capacity to regenerate ([1-3]) and has become a model organism for the functional analysis of the genes involved in pattern formation (e.g., [4]). Nonetheless, little is known about its evolutionary history and demographics, although it possesses several intriguing features. The genus *Schmidtea *includes only four species: *S. polychroa*, *S. lugubris*, *S. mediterranea *and *S. nova*. With the exception of *S. polychroa*, which has either amphimictic diploid and/or parthenogenetic polyploid populations, most of the *Schmidtea *species are amphimictic diploids. In *S. mediterranea*, in addition to the more common sexual amphimictic diploids, a third type of reproduction, asexual fissiparity [5], occurs in diploid populations bearing a heteromorphic translocation between 1^st ^and 3^rd ^chromosomes (only one chromosome of each pair is affected by the translocation) [6-8]. Although fissiparity is also common in other genera of planarians, such as *Girardia *[9] and *Dugesia *[10], most asexual fissiparous populations of these genera are triploid.

**Figure 1:**
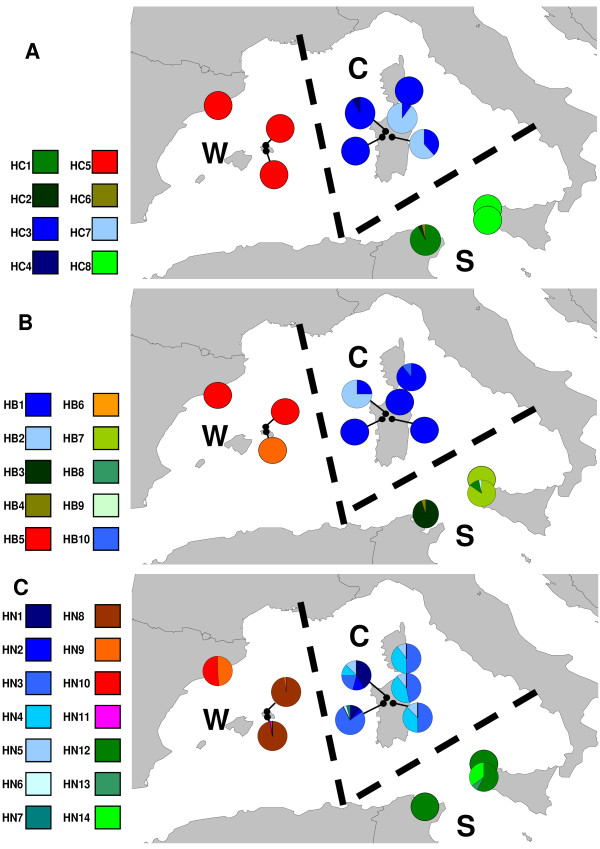
**Distribution of the species and haplotype distribution in populations**. Each map corresponds to a single gene: *COI *(A), *CYB *(B) and *N13 *(C). Pie charts indicate the proportion of the different haplotypes in each of the eleven populations. Colours and names of the haplotypes correspond to those indicated in the haplotype networks (Figure 2). Dotted lines show the geographical regions obtained in the SAMOVA analysis for 3 groups: WEST (W), CENTRAL (C) and SOUTHEAST (S).

*S. mediterranea *is restricted to the western Mediterranean in several scattered populations along the Catalan coast, Menorca, Mallorca, Corsica, Sardinia, Sicily and Tunisia [6-8,11,12] (Figure 1). The asexual strain occurs only in a few locations, in Catalonia and the Balearic Islands, where sexual populations have not yet been found. In the remaining distribution area, only sexual diploids are found, with the only exceptions being a triploid sexual population in Sardinia [13] and a triploid asexual (fissiparous) population in Menorca, the latter bearing a translocation between the 1^st ^and 3^rd ^chromosomes (only one of each triplet) [7].

This peculiar geographical distribution prompted Baguñà et al. [14] and De Vries et al. [11] to propose, according to the known geological history of the area at the time, that this was the result of microplate fragmentation and migration during the formation of the western Mediterranean basin. Indeed, the fragmentation of continental microplates (the Corsica-Sardinia block, CSb) and their clockwise and counterclockwise migration gave rise, during the Oligocene and early Miocene periods, to the Balearic Islands, Corsica, Sardinia, Calabria and the Kabilies in Algeria [15-18]. If this hypothesis is true, it implies that this species, or a close ancestor, was already present on the microplate before it first broke away, approximately 30 mya, splitting the CSb from the Spanish coast. The poor dispersion capability of freshwater planarians (they require permanent freshwater courses or ponds, and no forms resistant to desiccation have been described) and their impossibility to cross large marine masses, make other explanations (e.g., human or animal transport or recent colonisation) less plausible. Although a clear link between CSb fragmentation and migration and the successive speciation events has been demonstrated for other groups of animals [19-21], the case of *S. mediterranea *is unique because a correlation between geological events and its distribution has not been linked to any speciation event, which implies a more ancient origin for *S. mediterranea*.

Furthermore, when and how the asexual populations of *S. mediterranea *originated is also of considerable interest because no conspecific sexual individuals live in the same localities, the nearest populations being found in Sardinia and Corsica. This situation suggests multiple possible explanations for when and where they originated. Asexual *S. mediterranea *could have originated from Sardinian sexual populations a long time ago and remained isolated in the current area of distribution; they could have originated recently and have been transported by humans; or they could have a recent or ancient origin from sexual Spanish populations that are now extinct. Altogether, these ideas pose several interesting questions: 1) Is the microplate fragmentation the origin of the present distribution of *S. mediterranea*? 2) What is the age of this species? 3) When and where did the asexual strain appear? 4) Which sexual population is closest to the asexual ones? 5) Did the new asexual populations outcompete the original sexual populations, or did they originate in other regions and were brought to their current localities by human transport?

To answer these questions and to widen our knowledge on the evolutionary history of *S. mediterranea*, a well-resolved phylogeny is first needed. Molecular phylogenies are currently the best option to provide solid evidence on the origin, relationships and divergence in time among populations, particularly for organisms such as planarians, which are lacking reliable morphological characteristics. Furthermore, if these events can be correlated to some geological phenomena, a molecular clock can be set and calibrated, and the evolutionary history can be put into a temporal context. Finally, a comparative analysis of the genetic (nucleotide) variability within and among populations may allow us to identify the evolutionary factors responsible for the levels and patterns of genetic variation and therefore for the biological diversity [22].

We have sequenced fragments of two mitochondrial genes (one for the first time in planarians), and taking into account the genomic information available for *S. mediterranea*, we developed a new nuclear marker for planarians. Because nuclear and mitochondrial markers accumulate changes at different rates, they provide complementary information on the effects of different types of factors, namely, those affecting different historical times. Using these markers, we measured the levels of genetic variation among and within all populations sampled and inferred the phylogenetic tree for these species. In addition, we have calibrated, for the first time in planarians, a molecular clock to estimate the age of the species and the divergence among populations. The results obtained show the usefulness of the new markers and attest that the phylogeography and evolutionary history of *S. mediterranea *is far more complex than formerly envisaged.

## Methods

### Sampling, DNA extraction, gene amplification and sequencing

Samples covering all known distributions of *S. mediterranea *(Table 1) were collected between 2006 and 2009. Described populations from Mallorca [23] and north of Catalonia [7] could not be recovered. Sampling in Morocco and Algeria retrieved a population of *S. polychroa*, but no *S. mediterranea *was found. The populations from Spain, Corsica and Tunisia are the only populations described in the literature for those regions and were found during the present project sampling; no new populations have been found. Populations collected from Tunisia, Corsica, Sardinia and Sicily were identified as *S. mediterranea *by karyological analyses using standard procedures [24]. Altogether, a total of 217 specimens were collected from 11 localities. High molecular weight DNA was purified from live or ethanol-fixed specimens individually using DNAzol reagent (Molecular Research Center Inc, Cincinnati, OH). Specific primers (Table 2), some designed for this study, were used to amplify partial fragments of the mitochondrial genes *cytochrome oxidase I *(*COI*) and *cytochrome b *(*CYB*). We also searched for new nuclear markers, and based on sequences provided to us by other researchers (Abril, J., Adell, T. and Cebrià, F.), we designed primers to amplify and perform sequence analyses of different introns from the genes *glycogen synthase kinase 3 *and the *netrin receptor-like protein *(*netR*). The intron of the *glycogen synthase kinase *3 gene was very short (~ 50 bp) and showed low variability; thus, only a partial region of the 13^th ^intron of the *netrin receptor-like protein gene *(*N13*) was selected because it is the only fragment with the length and variability adequate for the proposed studies. Amplification products were sequenced directly with the same primers used in the PCR reaction using Big Dye (3.1, Applied Biosystems, Norwalk, CT, USA) and an automated sequencer, ABI Prism 3730 Applied Biosystems/Hitachi (Unitat de Genòmica dels Serveis Científico-Tècnics de la UB). DNA sequences were aligned with ClustalW [25] and optimised by sight using the amino acidic sequence as a guide for mitochondrial genes.

**Table 1:** Information on the studied populations

Population Code	Locality	Country	Sampling Date	Collector(s)	Reproduction	Ploidy	Translocation
*BAR_MON*	Montjuic, Barcelona	Spain	June 2009	Lázaro, E., Riutort, M., Vila-Farré, M.	Fissiparous	2n	yes
*MEN_GOR*	Gorg, Menorca	Spain	April 2007	Pons, S., Pretus, J.	Fissiparous	2n	yes
*MEN_MER*	Mercadal, Menorca	Spain	May 2007	Pons, S.	Fissiparous	3n	yes
*SAR_AST*	Astimini, Sardinia	Italy	November 2006	Pala, M.	Sexual	2n	no
*SAR_VAL*	Valverde, Sardinia	Italy	November 2006	Pala, M.	Sexual	2n	no
*SAR_CAR*	Carrabuffas, Sardinia	Italy	November 2006	Pala, M.	Sexual	2n	no
*SAR_SIL*	Silis, Sardinia	Italy	February 2006	Pala, M.	Sexual	2n	no
*COR_CAN*	Canella, Corsica	France	May 2009	Lázaro, E., Pala, M., Stocchino, G. A.	Sexual	2n	no
*TUN_LEB*	Lebna	Tunisia	November 2007	Harrath, A. H., Sluys, R.	Sexual	2n	no
*SIC_MAZ*	Mazaro, Sicily	Italy	May 2009	Lázaro, E., Pala, M., Stocchino, G. A.	Sexual	2n	no
*SIC_MAR*	Marausa, Sicily	Italy	May2009	Lázaro, E., Pala, M., Stocchino, G. A.	Sexual	2n	no

Origin of the populations, date of capture and collectors, type of reproduction, ploidy and the presence/absence of a heteromorphic translocation between chromosomes 1 and 3.

**Table 2:** Primers used in this study

Name	Sequence 5'-3'	Annealing Temperature (ºC)	Source
*COI*			
COIF (F)	CCNGGDTTTGGDATDRTWTCWCA	45	Lázaro et al., 2009
COIR (R)	CCWGTYARMCCHCCWAYAGTAAA	45	Lázaro et al., 2009
Bar2 (F)	CGTTTAGAGYTNTCTGTTCCAGG	40	Lázaro et al., 2009
COIbarc_plat_R (R)	TAATTAAAATATAAACCTCAGGATG	40	Lázaro et al., 2009
BarT	ATGACDGCSCATGGTTTAATAATGAT	43	Álvarez-Presas et al., 2011
*CYB*			
B3 (F)	TKRTWNTTCAGDTTKTTTCTGG	44	This study
B2 (R)	AAAATAYCACTCNGGCTTWAT	44	This study
*N13*			
MS13Fi	GGTAGTTGCATAAATTAAAA	50	This study
N13R2	GCTGAGAAACGGAAGCAAATCGAAGGG	50	This study

F, forward; R, reverse.

### Cloning

Some individuals were heterozygous for the nuclear gene *N13 *(Table 3). The haplotype phases were reconstructed using the PHASE algorithm [26,27] included in DnaSP v5 [28]. The PCR products of six individuals, two belonging to the *BAR_MON *population and four from the *SIC_MAZ *population, were cloned and sequenced to test the conflicting haplotypes obtained with PHASE. The PCR products were purified with a vacuum manifold and then cloned using the HTP TOPO TA Cloning Kit for sequencing (Invitrogen, California, USA). Eight colonies from each individual were amplified and sequenced using the T3 and T7 primers (included in the kit) following the procedure described in the previous section.

**Table 3:** Haplotype, genotype and nucleotide diversity values in populations and groups for each gene

		*COI *(699bp)					*CYB *(598bp)					*N13 *(336bp)								
	**Population**	***N*_T_**	***h***	***S***	***H*_D_**	***π***	***N*_T_**	***h***	***S***	***H*_D_**	***π***	***N*_T_**	***N*_HT_**	***N*_P_**	***h***	***S***	***H*_D_**	***π***	***g***	***G*_D_**

	*BAR_MON*	35	1	0	0	0	35	1	0	0	0	33	32	66	2	2	0.507	0.0030	2	0.061
W	*MEN_GOR*	19	1	0	0	0	19	1	0	0	0	19	1	38	2	1	0.053	0.0002	2	0.105
	*MEN_MER*	20	1	0	0	0	20	1	0	0	0	18	1	36	2	1	0.056	0.0002	2	0.111

	*SAR_AST*	14	1	0	0	0	13	1	0	0	0	13	2	26	5	6	0.406	0.0051	4	0.423
	*SAR_VAL*	20	2	1	0.189	0.0003	17	1	0	0	0	14	4	28	3	3	0.611	0.0046	4	0.736
C	*SAR_CAR*	13	2	1	0.513	0.0007	4	1	0	0	0	4	1	8	3	3	0.679	0.0049	3	0.833
	*SAR_SIL*	12	2	1	0.167	0.0002	12	2	1	0.409	0.0007	12	9	24	5	7	0.768	0.0103	5	0.833
	*COR_CAN*	11	1	0	0	0	10	2	1	0.200	0.0003	10	2	20	3	3	0.611	0.0046	3	0.689

	*TUN_LEB*	25	3	2	0.157	0.0002	18	2	1	0.111	0.0002	8	0	16	1	0	0	0	1	0
S	*SIC_MAZ*	28	1	0	0	0	27	2	1	0.271	0.0029	27	16	54	3	5	0.551	0.0060	5	0.715
	*SIC_MAR*	19	1	0	0	0	18	1	0	0	0	19	0	38	1	0	0	0	1	0

	WEST	74	1	0	0	0	74	2	1	0.400	0.0006	70	34	140	4	3	0.625	0.0022	5	0.548
	CENTRAL	70	4	3	0.509	0.0007	56	3	2	0.305	0.0005	53	18	106	7	7	0.682	0.0071	9	0.746
	SOUTHEAST	72	4	3	0.478	0.0007	63	5	9	0.509	0.0038	54	16	108	3	5	0.352	0.0037	5	0.534

	TOTAL	216	8	37	0.773	0.0223	193	10	28	0.803	0.0168	177	68	354	14	15	0.853	0.0081	19	0.865

*N*_T_, number of sequences; *N*_HT_, number of heterozygous individuals for *N13*; *N*_P_, number of sequences after haplotype reconstruction; *h*, number of haplotypes; *S*, number of polymorphic sites; *H*_D_, haplotype diversity; π, nucleotide diversity; *g*, number of genotypes; *G*_D_, genotype diversity; W, west group; C, central group; S, southeast group.

### Population genetics analyses

We used the program DnaSP v5.10 [28] to conduct most of the population genetic analyses. Haplotype diversity (*H*_D_) and nucleotide diversity (*π*) [29] were estimated for every population and gene. Genotype diversity for *N13 *was calculated by using the *H*_D _formula [29] and replacing haplotypes with genotypes. The *COI*, *CYB *and *N13 *haplotype networks were constructed using TCS v1.21 [30]. We used Spatial Analysis of Molecular Variance (SAMOVA v1.0 [31]) to define groups of populations, and we tested every gene for 2, 3 and 4 groups. This method is based on a simulated annealing procedure aimed at identifying groups of populations that are geographically homogeneous and maximally differentiated in terms of an among-group component of the overall genetic variance without the prior assumption of group composition that is necessary for AMOVA. The levels of divergence between *S. polychroa *and *S. mediterranea *and within and between the new groups were estimated using the *D*_xy _parameter [29]. The non-coding region of *N13 *presented high sequence variability between *S. polychroa *and *S. mediterranea*, so only a fragment of 191 bp could be unambiguously aligned to calculate the divergence (*K*) between the two species. Gene flow statistics *F*_ST _and the corresponding *Nm *were estimated for the three regions defined by SAMOVA. A genetic differentiation statistic, *S*_nn_, was estimated, and its statistical significance was determined using a permutation test (10,000 replicates). To determine if the pattern of the polymorphism for the *N13 *fragment conforms to that expected under the neutral hypothesis, we applied two neutrality tests to detect the specific fingerprint of recent population expansions, heavy bottlenecks or other selective and demographic scenarios: Tajima's *D *[32] and *R*_2 _[33]. These statistics were selected because they are not sensitive to the haplotype phase reconstruction and *R*_2 _has the advantage of behaving well for small sample sizes. Their statistical significance was estimated using coalescent computer simulations (10,000 replicates).

### Phylogeny and the molecular clock

All maximum likelihood (ML) phylogenetic analyses were determined using PHYML 3.0 [34] with the HKY model and 1000 bootstrap replicates. Bayesian inference (BI) was estimated using either with MrBayes 3.1 [35,36] or BEAST 1.6.1 [37].

Because the fossil record of freshwater planarians is non-existent, no molecular clock for the Tricladida is available. Hence, to calibrate the molecular clock and to infer the approximate age of *Schmidtea mediterranea*, we used two-step dating. First, an analysis with a partial sequence of the *COI *gene (309 bp), which belongs to 19 species of the families Dugesiidae and Planariidae (Additional file 1), was performed. Before running the dating analysis, we ran phylogenetic analyses for the short *COI *fragment using ML and BI (MrBayes, GTR with InvGamma, 10^6 ^generations with sampling every 10^3 ^and a burnin value of 10%) to test its resolution capability from the family to the species level. Then, we used BEAST with the following conditions: relaxed clock estimate, HKY evolutionary model and Yule process speciation, during 10^7 ^generations with sampling parameters every 10^3 ^and a 10% burnin value for the final trees, as recommended in the BEAST manual. We calibrated the data based on the biogeographical hypothesis proposed by Ball (1974) [38]. Analysing the present day distribution of the Dugesidae genera, Ball suggested that the center of the origin of this family would have been situated in southern Gondwanaland. The breakage of this supercontinent, thus separating from what would become America to the west, would have resulted in the origin of the *Girardia *genus (exclusively American) in that region, while the rest of the genera (or their ancestors) would have appeared in the eastern block; *Dugesia *and *Schmidtea *(currently present in Africa, Asia and Europe) would have originated in Africa and Europe, respectively, after their ancestors moved northward. Hence, we have used the split between the continents of Africa and South America (ending around 100 mya) as a calibration point to determine the maximum age of the separation of the genera *Dugesia *and *Schmidtea *from the genus *Girardia*.

In the second dating step, we used 192 sequences from the *COI *(920 bp) and *CYB *(677 bp) genes of *S. mediterranea *and the mean rate value obtained for *COI *in the previous analysis to determine the age of separation between the populations of *S. mediterranea*. For this second analysis, which consisted of a comparison of populations from the same species, it was expected that the rates of evolution would not vary among populations. Hence, the conditions for the analysis were the following: strict clock, HKY evolutionary model, coalescent model with constant population size for 10^8 ^generations, sampling every 10^4 ^generations and a burnin of 10%. In addition, a phylogenetic analysis with ML was performed to obtain boostrap support for the nodes.

## Results

### Datasets

For the *COI *and *CYB *sequences, we created two datasets for each gene: one for phylogenetic analyses and the other for population genetic studies. For phylogenetic analyses, the *COI *alignment contained 216 sequences of 920 bp. *CYB *was more difficult to amplify, so the dataset contained only 193 sequences of 677 bp. However, the ends of some sequences, for both *COI *and *CYB*, were shorter because of sequencing difficulties and contained a series of Ns. Additionally, some *COI *gene sequences were amplified in two non-overlapping fragments, and this procedure resulted in a short region between them (~50 bp) in which some individuals had unknown nucleotides. In both cases, these sites were removed from the datasets for the rest of analyses. This removal reduced the length of the *COI *dataset to 699 bp and of the *CYB *dataset to 598 bp. The nuclear fragment (*N13*, a non-coding region) was used in population genetic analyses but not in phylogenetic inference analyses because its levels of polymorphism were high within the populations studied but low among them and, consequently, there was not enough information for the analyses at this level. Additionally, there were difficulties with amplification of *N13 *in some individuals and, hence, a single dataset was obtained with 177 sequences of 336 bp.

### Levels and patterns of nucleotide diversity

All nucleotide polymorphism results are shown in Table 3. We found 8 haplotypes in the 216 *COI *sequences analysed [GenBank: JF837055 to JF837062] and 10 haplotypes in the 193 *CYB *sequences [GenBank: JF837045 to JF837054]. For both genes, a single haplotype was found in 7 of the 11 populations studied. For *N13*, we found only two populations with a single allele, *TUN_LEB *and *SIC_MAR*, because the remaining populations had more than one allele and at least one heterozygous individual [GenBank: JF837063 to JF837081]. We used the PHASE algorithm to reconstruct the alleles of *N13 *that were present in the heterozygous individuals in these populations. PHASE results showed that the two populations with more heterozygous individuals (*BAR_MON *and *SIC_MAZ*) shared an allele. Because these two populations are geographically distant and differ in the presence/absence of the translocation and in their type of reproduction, we decided to clone the *N13 *PCR product of some individuals of each population to verify the existence of this common allele. The sequences obtained (Additional file 2) showed that *BAR_MON *and *SIC_MAZ *do not share any allele. The conflicting results between the PHASE outcome and the cloning experiments were most likely derived from the assumption of PHASE in which all of the individuals analysed belong to a single panmictic population in Hardy-Weinberg equilibrium, a situation that posterior analyses (see below) showed not to be the case here. Finally, for the 177 individuals analysed, after phase reconstruction and cloning verification, we found 14 alleles in the 354 *N13 *sequences.

Taking all of the populations together, the haplotype diversity values were high and similar for the three genes (0.773, 0.803 and 0.853), whereas nucleotide diversity was much higher in the mitochondrial genes (0.0223 and 0.0168) than for the nuclear marker (0.0081). Instead, haplotype and nucleotide diversity within the populations were lower for the mitochondrial genes than for the nuclear fragment (Table 3).

The nuclear gene *N13 *showed very similar values of haplotype and nucleotide diversity within the populations (Table 3), with the only exception being the two Menorca asexual populations, which had nearly no variability. Additionally, the genotypic variability was also very low for the three asexual populations.

### Geographical division and genetic differentiation among and within geographical groups

**Figure 2:**
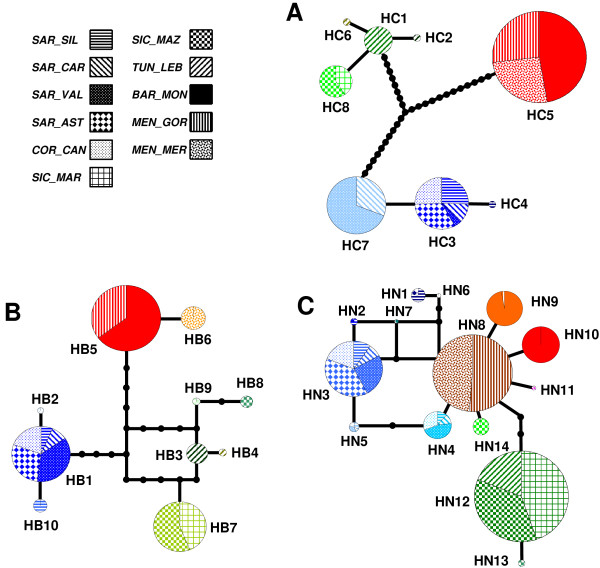
**Gene haplotype networks**. Each network corresponds to a single gene: *COI *(A), *CYB *(B) and *N13 *(C). Different textures represent the studied populations, and each coloured circle represents a haplotype found in the sample. Black dots represent intermediate (non-present) haplotypes, lines connecting haplotypes (either existing or not) represent one nucleotide change, the size of each circle is proportional to the haplotype frequency in the sample and the colours of the circles correspond to the three groups: W (red), C (blue) and S (green).

*COI *and *CYB *haplotype networks (Figure 2A and 2B) show three well-differentiated groups that match the current geographical distribution of populations (west, central and southeast of the known distribution) (Figures 1A and 1B). The *N13 *network does not at first glance show a clear division among the haplotypes (Figure 2C). However, after using SAMOVA (Additional file 3) for *N13*, we found three groups of populations with the same clustering as was found for the mitochondrial genes (Figure 1C). Accordingly, the groups obtained were named WEST (W, populations from Barcelona and Menorca), CENTRAL (C, populations from Sardinia and Corsica) and SOUTHEAST (S, populations from Sicily and Tunis). When these groups were analysed using the *S*_nn _statistical test, they were found to be significantly different. Moreover, the *F*_ST _statistic was high, and the values for *Nm *were very low for the three genes (Table 4).

**Table 4:** Gene flow and genetic differentiation analyses among geographical groups

	*COI*	*CYB*	*N13*
N ind	216	193	354
*F*_ST_	0.9853	0.9406	0.5882
*Nm*	0.01	0.03	0.18
*S*_nn_	1	1	1
*S*_nn _p-value	0*	0*	0*

*Significant differences. High *F*_ST _and low *Nm *values suggest that there is no gene flow among the three groups. Significant differences in the *S*_nn _statistic shows the same result.

For the mitochondrial genes (Table 3), the groups presented low nucleotide diversity (π) with the sole exception of *CYB *from the S group. In some cases (S group *COI*, W and S *CYB*), the values of mitochondrial haplotype diversity (*H*_D_) for the groups were higher than those observed for the individual populations within them, which is indicative of differentiation among their populations. The values of nucleotide and haplotype diversity for *N13 *were, in general, higher than those for the mitochondrial genes.

The average number of nucleotide substitutions per site (*K*) between *S. polychroa *and *S. mediterranea *were similar for the three genes (15% for *COI*, 17% for *CYB *and 14% for *N13*; Table 5). For mitochondrial genes, *D*_xy _values between populations and within each group (W, C and S) were much lower (at least four times lower) than among groups (Table 5). The highest *D*_xy _values among groups were situated at approximately 3.5%, demonstrating a large gap between the higher intraspecific distances and the mean interspecific values (15-17%). For *N13*, this gap was more evident because the higher interpopulation distances were 1.4%.

**Table 5:** *D*_xy _for each gene among and within groups and *K *between *S. mediterranea *and *S. polychroa*

*COI*	W	C	S
WEST	0.000		
CENTRAL	0.035	0.001	
SOUTHEAST	0.032	0.032	0.001
*S. polychroa*	0.153	0.155	0.141

*CYB*			

WEST	0.001		
CENTRAL	0.028	0.001	
SOUTHEAST	0.028	0.016	0.004
*S. polychroa*	0.176	0.178	0.166

*N13*			

WEST	0.002		
CENTRAL	0.007	0.007	
SOUTHEAST	0.009	0.014	0.003
*S. polychroa*	0.135*	0.137*	0.148*

*K calculated from a fragment of 191 bp.

### Demography analysis and neutrality test

Neutrality tests, based on Tajima's *D*, showed significant positive values (Table 6) in four populations: *BAR_MON *(W group) and in three populations of the C group (*SAR_VAL*, *SAR_SIL *and *COR_CAN*), whereas the *SAR_CAR *population was also very close to the right 95% confidence interval. Ramos-Onsins and Rozas' *R_2 _*statistical test showed significant values for the same populations and also for the single population in the S group with more than one haplotype (*SIC_MAZ*). However, Menorcan populations (W group) showed negative, although not significant, values for Tajima's *D*.

**Table 6:** Neutrality test results

		Tajima's *D*		Ramos-Onsins & Rozas *R*_2_	
		*D*	95% CI	*R*_2_	95% CI
	*BAR_MON*	2.3662*	(-1.5574, 2.0410)	0.2536*	(0.0429, 0.2124)
W	*MEN_GOR*	-1.1286	(-1.6948, 1.8107)	0.1601	(0.0601, 0.1782)
	*MEN_MER*	-1.1332	(-1.6847, 1.8405)	0.1643	(0.0609, 0.1785)

	*SAR_AST*	0.2722	(-1.7092, 1.9445)	0.1431	(0.0739, 0.2206)
	*SAR_VAL*	2.3691*	(-1.7208, 1.9355)	0.2553*	(0.0719, 0.2262)
C	*SAR_CAR*	1.7278	(-1.5952, 1.7641)	0.2738	(0.1339, 0.3307)
	*SAR_SIL*	2.6559*	(-1.7501, 1.7979)	0.2477*	(0.0735, 0.2052)
	*COR_CAN*	2.1833*	(-1.7233, 1.9217)	0.1999*	(0.0504, 0.1887)

	*TUN_LEB*^1^	x	x	x	x
S	*SIC_MAZ*	1.9595	(-1.6633, 2.0016)	0.2561*	(0.0883, 0.2363)
	*SIC_MAR*^1^	x	x	x	x

W, west group; C, central group; S, southeast group. *Significant differences. ^1^These populations had only one haplotype. Significant differences in some populations suggest that the variability observed cannot be explained by the neutral hypothesis.

### Phylogeny and the molecular clock

To test the phylogenetic informativeness of the short *COI *gene fragment before dating analysis, two phylogenetic analyses by ML and BI were performed. Both resulting trees were concordant with the accepted phylogeny for freshwater planarians [38-40] (support values shown in the dating tree are in Additional file 4).

The analysis with BEAST of these data placed the time of the most recent common ancestor between *S. mediterranea *and *S. polychroa *at 43 mya (between 72.23 and 24.96 mya, Additional file 4) and the time of the most recent common ancestor (TMRCA) of *S. mediterranea *between 19.55 and 5.9 mya. The resulting dates for the divergence of *Dugesia cretica *from the other two Greek species (*D. ariadnae *and *Dugesia *sp. from Peloponnese) within *Dugesia *and the divergence among occidental and oriental *Dugesia *species are congruent with a geologically based biogeographical hypothesis [41,42]. Finally, the average rate of evolution calculated for the *COI *gene by BEAST for the whole tree is 0.0027 mutations per site per million years (0.27% substitutions per million years), which implies a sequence divergence of 0.54% per million years. This is between one-half and one-fourth of the rate of 1-2%, which is commonly used as a standard for mitochondrial genes (mainly derived from arthropods [43]) in many studies. However, similar low values have been found in other groups, e.g., 0.67-0.95% sequence divergence in molluscs of the Arcidae family [44]. While planarians have some of the lowest rates of substitution, this does not invalidate the dating results obtained.

**Figure 3:**
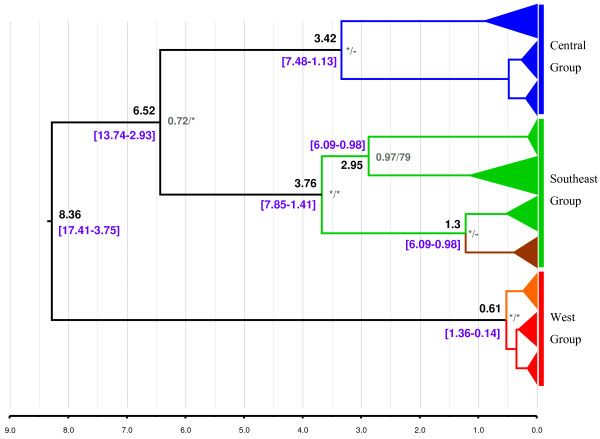
**Ultrametric tree obtained with BEAST combining *COI *and *CYB *datasets for all individuals**. Black numbers represent the age of the nodes, and purple numbers represent their confidence intervals (95%). Grey numbers represent the posterior probability/bootstrap values. * indicates the maximum value; - values <0.5 or 50%. The *MEN_MER *population is in orange, and the *TUN_LEB *population is in brown.

Using the mean rate obtained in the previous analysis (0.0027), we performed the second dating analysis with the two mitochondrial genes. The analysis included all individuals for which we obtained sequences of both mitochondrial genes. The topology of the resulting tree within *S. mediterranea *was the same as that obtained in the previous analysis, and node ages and dating intervals between the three groups were consistent in both analyses (Figure 3 and Additional file 4). W, C and S groups were monophyletic with high posterior probabilities in the Bayesian analysis, and western populations appeared to be the basal group of the three groups. The populations within the regions did not constitute monophyletic groups, with two exceptions: the individuals from *TUN_LEB *(brown in Figure 3) constitute a monophyletic clade (1 PP/79 BS) buried within the S group, and this clade is a sister clade to some individuals from *SIC_MAZ*; and the individuals from *MEN_MER *(orange in Figure 3) also constitute a monophyletic clade (1 PP/65 BS) that is a sister clade to a clade that includes a mix of individuals from the other two populations of the W group.

## Discussion

### Is *Schmidtea mediterranea *a single species?

The levels of genetic divergence found among all of the populations of *S. mediterranea *studied, which cover most of its current range of distribution, suggest that it is a single species. This suggestion is based on the large gap between the higher genetic distance values obtained among *S. mediterranea *populations and those between the *S. mediterranea *populations and *S. polychroa *(values of 0.035 for *COI*, 0.028 for *CYB *and 0.014 for *N13 vs. *the means of 0.15, 0.17 and 0.14, respectively). Furthermore, these values are within the range for other Tricladida species. In *Dugesia *and *Microplana *(a freshwater and a terrestrial genus, respectively), the *COI *divergence within species is less than 0.04, whereas among species it is greater than 0.10-0.12 [10,45]. Such differences are clearly visualised in the first phylogenetic dating analysis, which includes the other three *Schmidtea *species (Additional file 4).

*S. mediterranea *is genetically structured. This is evident for the mitochondrial genes and, although not as clearly, for the nuclear intron as well. The haplotype networks for both mitochondrial genes show three well-defined groups that are separated by a similar number of differences, and these groups correspond to the same three clades separated by deep nodes in the phylogenetic analyses (Figures 2 and 3 and Additional file 3). The structure shown by the haplotype networks and phylogenetic trees is confirmed by the SAMOVA results, which show that the same three groups are the ones that best explain the genetic structure in relation to geographical distribution, in this case, for the three markers (Additional file 3). Additionally, genetic differentiation analyses (Table 4) show that the three groups are significantly differentiated both for the mitochondrial and nuclear genes (high *F*_ST _values and a significant *S*_nn _value). The differences in genetic diversity patterns found between nuclear and mitochondrial markers generally result from differences in coalescence times between the two genomes. The fact that in certain analyses the nuclear marker also detects the division into three groups supports the idea that this might be the result of an old isolation; together with the observed lack of gene flow among populations, this finding could indicate that these groups are undergoing speciation or even constitute cryptic species (although the reported inter-fertility among C and S individuals [46] makes this latter hypothesis less plausible).

### The geographical distribution of *Schmidtea mediterranea*: remnant of an old distribution driven by microplate dispersal or indicator of recent colonisations?

The genetic structure found for *S. mediterranea *does not reflect a process of recent colonisation from one region to the others. Indeed, the genetic pattern found -- with significant differentiation among geographical groups and even among populations within each group (polymorphism parameters show low levels of polymorphism within populations but high levels when all populations are considered together for mitochondrial genes, which reflects high differentiation among the populations) --indicates better an ancient differentiation among these three geographical groups and even within them. Moreover, the shape of the haplotype networks, which shows the groups to be equidistant, suggests that no region is central to the others. Altogether, these findings strongly suggest that the current distribution of *S. mediterranea *is a relic of the split from a former larger geographical distribution.

Taking into account the phylogeny of *S. mediterranea*, could the fragmentation and migration of microplates be the sole reason for its current distribution? Despite the evident complexities of the geology of the western Mediterranean [16,17,47], there is a general agreement on the original location of the microplates of Corsica, Sardinia, part of Calabria, the Balearic Islands and both Kabylies (Grande and Petite), which form the CSb, the region that separated from Iberia and Eurasia 30 mya. In the first event, the southern assemblage, which included the Balearic Islands and the Grande Kabylie, broke off and moved clockwise from Iberia, and the northern assemblage, which included Corsica, Sardinia, part of Calabria and the Petite Kabylie, broke off and moved counter clockwise from Eurasia. Next, the crucial event was the breakage between both assemblages that gave rise to the Sardinian Channel at 24 mya, dividing the western from the central and southern areas. Between 16 mya and 10 mya, Corsica and Sardinia reached their present locations and collided with future peninsular Italy (then connected with Sicily). Finally, the Tyrrhenian Sea opened between 8.6 mya and 7.6 mya [47]. In parallel, the Grande and Petite Kabylies broke off from the Balearic Island and Sardinia microplates, respectively, between 25 mya and 20 mya, moved southwards and collided with Africa to reach their final positions approximately 15 mya. Furthermore, the history of the islands, such as the Balearic Islands and Sicily, is even more complex because in the last 20 my, they have endured periods of submersion and emersion, as well as continental contacts. Thus, ~14.8 mya, as attested by the entrance of diverse animal species, Menorca and Mallorca were in contact with the Iberian Peninsula. Later, a marine transgression covered both islands, wiping out most of the Protoligurian fauna and flora, only sparing some species in the highest parts of Mallorca (Serra de Tramuntana). A second contact with the peninsula, approximately 5 mya (during the Messinian crisis, when the sea level dropped dramatically), allowed the entrance of some mammal species. Finally, during the Pleistocene glaciations, the two islands formed a single landmass, easing the migration of the remaining Protoligurian and other fauna from Mallorca to Menorca [48,49].

The first dating analysis (Additional file 4) provides an age of ~43 mya for the divergence of *S. mediterranea *from its sister species *Schmidtea polychroa*. This date and the age inferred for the older node of *S. mediterranea *in our two dating analyses (between ~20 and ~5 mya; Figure 3, Additional file 4) sets a time range for the origin of the species that is congruent with the hypothesis that *S. mediterranea*, or its most recent ancestor, was already present on the CSb microplate (or microplates [18]) when the microplate broke off from the continent ~30 mya. Furthermore, the basal position of the W group (Spanish populations) is consistent with the next breakage of the CSb into the northern and southern microplate assemblages. Finally, the splitting of Corsica and Sardinia from Calabria during the opening of the Tyrrhenian Sea (7.6-8.6 mya) and the connection of the latter, via Sicily, with Tunisia would have resulted in the separation of the C and S groups (~7 my of age for the last common ancestor). The spreading of the groups from Calabria to Sicily and Tunisia was likely very hazardous given its low dispersal capability, but it happened during a rainier period than today. Indeed, the Mediterranean climate as we know it, with dry summers, did not appear until 3.2 mya [50] with the main difference being the frequency of rain, rather than the temperature, which should have resulted in more freshwater courses and, hence, more opportunities for freshwater planarians to disperse. Last but not least, with the present data, we can also postulate that the present asexual populations (W group) are descendants of the original sexual *S. mediterranea *that remained on the continent or in the Balearic Islands after Sardinia and Corsica broke away, although the dating obtained is too recent to be explained by the geological history (between ~20 and ~4 mya vs. 24 mya). Alternatively, this age could be consistent with a variation of the CSb migration hypothesis [51] in which the microplate including Corsica-Sardinia and Calabria remained connected to the Paleo-Europe continent during its migration, becoming detached ~5 mya, when the Mediterranean was refilled after the Messinian salinity crisis and simultaneously with the raising of Tuscany. This alternative could explain the closeness of the split between the three geographical groups in our phylogenetic trees and their equidistance in haplotype networks. However, in this case, we would expect *S. mediterranea *to be distributed across a wider area, including Liguria and the Mediterranean coast of France, but *S. mediterranea *has never been described in these places or been found there in recent samplings, whereas *S. polychroa *and other planarian species have. One can consider that the asexual character of the W group populations could have affected the dating analyses, resulting in artefactually younger datings (see the next section).

Compared to its sister genus *Dugesia *(with more than 20 species described in the Mediterranean), the genus *Schmidtea *(with a mere 4 species) has a much lower diversity (see Additional file 4). Even in the area where we find *S. mediterranea*, *Dugesia *has diversified into at least 4 species (*D. hepta*, *D. benazzii*, *D. subtentaculata*, and *D. liguriensis*) probably as a consequence of the same geological events described here. Therefore, the results point to *Schmidtea *as a low diversifying genus and to *S. mediterranea *as an old species compared to others in the same area, even within the Tricladida. With the exception of *S. mediterranea *and some populations of *S. polychroa *in North Africa and Sardinia, most *Schmidtea *inhabit continental Europe up to Scotland and Sweden. Sexual *S. polychroa *and *S. lugubris *lay cocoons between 10°C and 23-25 °C [52] because higher temperatures are deleterious. Even for sexual populations of *S. mediterranea*, Harrath et al. [12] showed a moderate to drastic reduction in the size of the testes, ovaries and the copulatory apparatus begins at over 20 °C and worsens over 25 °C. In other words, *Schmidtea *seem best adapted to climates with moderate summer temperatures, a situation that is by no means found on the Mediterranean islands and in continental coastal areas. This raises a final intriguing question: could *S. mediterranea *be considered a survivor trapped in a harsh habitat as a consequence of CSb breakage and migration?

### The asexual populations of *S. mediterranea*: Single or recurrent origins? Recent or ancient?

Genetic variability at the mitochondrial level is null within asexual populations and very low when the three populations are taken together (only for *CYB *did one of the three populations show a different haplotype diverging only at one nucleotide). In contrast, the nuclear marker *N13 *shows, namely, in the Barcelona population, high haplotype diversity similar to that found for sexual populations in other regions. This value might, however, be rather misleading because the Barcelona population is almost exclusively made up of heterozygous individuals (32 out of 33). When we measure genotype diversity instead of haplotype diversity (Table 3) all asexual populations show lower genetic variabilities than the sexual populations. In summary, whereas in sexual populations the variability is evenly distributed among all genotypes (whether heterozygous or homozygous), in asexual populations variability is exclusively found in specific genotypes. Although the latter situation would be at odds with a panmictic population, it might be anticipated for an asexual population that reproduces by fission.

How is the very low or null variability in asexual fissiparous *S. mediterranea *explained? Most likely, when a few individuals, or even one, became asexual within a sexual population (assuming that they persisted and outcompeted their sexual counterparts), this genotype would become fixed, which would result in the disappearance of the sexual individuals and of most genetic variability. Present climatic conditions and the types of habitat where these asexual forms are found make this scenario very likely. In Menorca, the asexual forms live in temporary water courses that become dry or are reduced to small ponds during summer, with running water only after rainfall in winter and early spring. Under these conditions and with a fair amount of food available, fissiparous organisms (which duplicate in number after fission and regeneration in 15- 20 days) could reproduce faster than sexual individuals, which have higher energy demands than asexual individuals because they need to develop and maintain reproductive organs, mate, and lay cocoons [53]. In fact, the negative values obtained in the Tajima's *D *neutrality test, although not significant, provide support for an expansion signal for the two Menorca populations. For the Barcelona population, the situation is different because it is localised in a plant nursery without seasonal water shortages. However, other factors, such as periodic pond cleaning in the plant nursery, may cause some decreases in population size. The significant positive values obtained in the neutrality tests may result from these types of recent and less pronounced bottleneck.

It is important to bear in mind that asexual reproduction by fissiparity poses problems that are very different from the much more common asexual reproduction by parthenogenesis. In parthenogenetic populations, new genetic variants could arise and spread by mutation in the germline, and with the occurrence of meiosis, new combinations could arise by recombination. In fissiparous populations, all mutations are somatic instead, and the probability of a new mutation spreading over a whole individual, and thereafter through the whole population, should be very low and could explain the extremely low rates of substitutions in these populations. Therefore, the genetic composition found in these organisms might be a frozen picture of the genetic pattern that was present in the first animals that became asexual. Although very low rates of substitution (absence of nucleotide diversity) are evident in the data presented here, more in depth studies with new markers are necessary to understand how these populations keep the burdens of a lack of genetic variability at bay.

From the data obtained, is it possible to make an educated guess about when and where asexual populations originated and spread? The close genetic relationship among the Menorca and Barcelona populations, the sharing of mitochondrial haplotypes, and their geographical closeness argues for a single asexualisation event. After the asexual individuals outcompete the original sexual population, they might have spread from the island to the continent or vice versa (the former is more likely given the basal situation of *MEN_MER *in the W clade) either by passive dispersal or through human activities, that has been proven for several species, freshwater planarians among them (Ribas *et al. *[9] for *Girardia tigrina*; and Lázaro *et al. *[10] for *Dugesia sicula*). However, although the estimated age for the clade, including the *BAR_MON *and *MEN_GOR *individuals, is too recent to be explained by the geological events described, it is also too old to be explained by human transport (0.6-0.2 mya). As for the age of onset of the asexual lineage, it is recent according to the point of diversification among fissiparous populations (1.36-0.14 mya).

However, this and the recent dating obtained for the basal splitting in the species tree (Figure 3) might be underestimates caused by the anomalously low genetic substitution rates of fissiparous organisms misleading the methodologies used in the dating analyses. To test this hypothesis further, we performed a test and attempted to model the change in the evolutionary rate in the asexual lineage by using BEAST. To do this, we repeated our second dating analysis, and this time we considered the asexual sequences as fossils so that at the point in the past where they first appear, the lineage stops accumulating changes (Additional file 5). The results show that, if we assign an old age to the asexuals (around 20 mya) and use the rate obtained in our first dating analysis, we recover splitting ages among geographical groups that are close to those that would be expected if the CSb hypothesis were true. The low substitution rates of fissiparous populations could spare them the burden of accumulating deleterious mutations and explain this longevity, although they still need to avoid the problem of a lack of variability. Further genetic analyses, including more coding regions, could help to ascertain whether these hypotheses are valid and to understand how mutations spread within individuals and became fixed in populations of these asexuals.

A final conundrum of the asexual populations of *S. mediterranea *is the presence of the fissiparous triploid population (*MEN_MER*) that bears the translocation in one of its three chromosomal sets. In freshwater planarians, triploid individuals are known to form in sexually reproducing populations (e.g., in *S. polychroa *[54,55]) from the union of unreduced diploid ovules (or sperm) and haploid sperm (or ovules). The *MEN_MER *population bears two normal chromosomal sets with one set including the translocation between the 1^st ^and 3^rd ^chromosomes. Therefore, this population could have originated from the union between an unreduced diploid ovule from a sexual diploid and a sperm bearing the chromosomal translocation from an asexual individual. In laboratory cultures, large (e.g., >20-25 mm in length) individuals from asexual fissiparous populations often develop testes, large ovaries, and the whole copulatory complex [6,56] and have been reported to mate with sexual diploids, although the cocoons that are laid are usually sterile. Although fissiparous specimens do not usually reach this size in nature, such an event is the more reasonable explanation for the origin of fissiparous triploids in *S. mediterranea*.

### The sexual populations of *S. mediterranea*

The sexual populations studied showed extremely low mitochondrial variabilities (Table 3), with two of them (one in Sardinia, one in Sicily) having no variability. Data on intrapopulation variability for other triclads are scarce, but two terrestrial planarian species from the Atlantic forest in Brazil have notably higher values of π (0.01 and 0.017 for *COI *[57]). Although other groups of animals show very low values of π [e.g., *Palinurus elephas *(0.0013-0.0030 for *COI*, [58]); and the lepidopter *Helicoverpa armigera *(0.0017-0.0038 for *COI*, [59])], the values are not as low as those found for *S. mediterranea*. For the nuclear marker, intrapopulation variability is higher than for mitochondrial genes, although again two populations (from Tunisia and Sicily) show no variability. However, as pointed out above, the high diversity found among geographical regions, even for the nuclear marker, suggests an old origin for the present distribution of planarians and, hence, for their populations.

Because old populations are expected to contain high variability (because long stable populations accumulate changes), the low levels determined here could be explained by demographic events affecting neutrality. The neutrality tests applied (Table 6) show that many of these populations demonstrate a significant result against the hypothesis of neutrality, with the estimated values on the positive side of the interval obtained in the permutation test. This finding may be interpreted as a consequence of recent bottlenecks (that would also explain the significant genetic differentiation found among populations; data not shown). This interpretation is in agreement with the habitat and climate conditions in which these populations live, even though their demographic conditions are not as harsh as those for the fissiparous populations from Menorca, and the sexual character of the C and S populations results in a different outcome (here, there are no signs of population expansion).

The relationship between Sicilian and Tunisian populations merits a final comment. The close relationship between the Tunisian population and some individuals from the Sicilian populations may indicate that the only Tunisian population (despite extensive sampling [12,60]) was recently introduced by human activities. However, this is unlikely because the Sicilian and Tunisian populations do not share a mitochondrial haplotype and because the age of the node giving rise to the Tunisian population (Figure 3) far exceeds the age in which human activities began in the Mediterranean. Therefore, the scarcity of *S. mediterranea *in Africa (it has so far not been reported from Algeria to the Canary Islands) may be explained by competition between the abundant fissiparous triploids of *D. sicula *[60] and *S. mediterranea*. Fissiparous populations of *D. sicula *are present all around the Mediterranean coastal areas (from Greece and Israel to the Canary Islands [10]; E. Solà and M. Riutort, unpublished data) where they seem well adapted to high temperatures and dry conditions [60,61]. It is not surprising that *D. sicula *may outcompete *S. mediterranea*, which at present has been reduced to a single locality found in Tunisia, despite a reasonable sampling coverage.

## Conclusions

*Schmidtea *is a genus with a low diversity that is constituted by only four species. These species are probably quite old (~40 mya) and primarily have a northern European distribution. They are probably better adapted to cold climates, which are less likely to allow speciation, than to hot climates. Ball [38] has suggested that *Schmidtea *species are in morphological stasis (differences in morphology among species are circumscribed to details of their reproductive organs), and the present study seems to indicate that the stasis is also found at the molecular level.

The present distribution of *S. mediterranea *is best explained, as suggested long ago, by a vicariant process of microplate fragmentation and migration of the CSb during the Oligocene and Miocene periods. Regardless of which of the two possible outcomes of this hypothesis better explains the distribution of *Schmidtea*, the age of the species is relatively old compared with the species of other groups present in the same area, even compared with its sister genus *Dugesia*. Asexual fissiparous populations of *S. mediterranea *probably arose within the west area; however, the lack of sexual populations in that area precludes a good estimate of whether it was an ancient or a recent event. Last but not least, the sparse number of localities where *S. mediterranea *is currently present in the continental range, i.e., on the Tunisian and the Catalan coast, suggests that *S. mediterranea *may be a survivor trapped in a harsh habitat after a long voyage to the South on several microplates after the breakage of the CSb 30 mya from which *S. mediterranea *has slowly been displaced by better-adapted species.

## Authors' contributions

JB and MR designed the study. AHH, EL, MR, MP and GAS collected samples. AHH, MP and GAS performed the karyological study. EL performed the molecular work and obtained the sequence data. EL and MR conducted all of the analyses, and JB, EL and MR wrote the first draft. All authors read and approved the final manuscript.

## Supplementary Material

Additional file 1 **Species used in the first dating tree and their GenBank Accession Numbers**. Click here for file

Additional file 2 **Netrin haplotypes obtained in the cloning process for each individual**. Click here for file

Additional file 3 **Results of the SAMOVA analysis**. **A**, W vs. C and S populations; **B**, W and C vs. S populations; **C**, W vs. C vs. S populations; **D**, W vs. C vs. *TUN_LEB *vs. Sicilian populations; **E**, W vs. S vs. *SAR_SIL *vs. C populations, except *SAR_SIL*. The percentage of variation among groups greatly increased in *COI *and *CYB *from 2 to 3 groups but remains almost unchanged from 3 to 4. Additionally, the percentage of variation among populations within groups greatly decreased in the three genes from 2 to 3 but decreased very little from 3 to 4. Click here for file

Additional file 4 **Ultrametric tree obtained with BEAST to find the age of the split between *S. mediterranea *and *S. polychroa***. Numbers in nodes represent the posterior probability/bootstrap values obtained in the phylogenetic analyses performed with MrBayes and PHYML (not shown). * indicates the maximum value, and the symbol - indicates values <0.5 or 50%. Purple numbers represent the confidence interval (95%) for the age of the node. Coloured branches indicate the membership of *S. mediterranea *individuals to one of the geographical groups: W in red, C in blue and S in green. Click here for file

Additional file 5 **The effect of asexual individuals on the dating analyses**. Click here for file
