# Supplementary material for: Schmidtea mediterranea phylogeography: an old species surviving on a few Mediterranean islands?
Source: BMC Evol Biol. 2011 Sep 26;11:274. doi: 10.1186/1471-2148-11-274 (PMC3203090; doi:10.1186/1471-2148-11-274)
Supplement: Additional file 1 — Species used in the first dating tree and their GenBank Accession Numbers. [file 1471-2148-11-274-S1.DOC]

| Species | Accession Number |
| --- | --- |
| Family Dugesiidae |  |
| *Dugesia* sp. (Peloponnese) | JN376140 |
| *Dugesia ariadnae* | JN376142 |
| *Dugesia cretica* | JN376141 |
| *Dugesia gonocephala* | DQ666033 |
| *Dugesia japonica* | DQ666034 |
| *Dugesia ryukyuensis* | AF178311 |
| *Dugesia sicula* | DQ666035 |
| *Dugesia subtentaculata* | DQ666036 |
| *Girardia dorotocephala* | AF178314 |
| *Girardia schubarti* | DQ666041 |
| *Girardia tigrina* | AF178316 |
| *Schmidtea lugubris* | AF290022 |
| *Schmidtea mediterrranea* | AF178322 |
| *Schmidtea nova* | AF290023 |
| *Schmidtea polychroa* | AF178323 |
| Family Planariidae |  |
| *Crenobia alpina* | AF178308 |
| *Phagocata* sp. | DQ666053 |
| *Polycelis felina* | DQ666049 |
| *Polycelis tenuis* | AF178321 |

**Additional file 1. Species used in the first dating tree and their GenBank Accession Numbers**
