# Supplementary material for: Schmidtea mediterranea phylogeography: an old species surviving on a few Mediterranean islands?
Source: BMC Evol Biol. 2011 Sep 26;11:274. doi: 10.1186/1471-2148-11-274 (PMC3203090; doi:10.1186/1471-2148-11-274)
Supplement: Additional file 2 — Netrin haplotypes obtained in the cloning process for each individual. [file 1471-2148-11-274-S2.DOC]

| Population | Individual code | HN9 | HN10 | HN12 | HN13 | HN14 |
| --- | --- | --- | --- | --- | --- | --- |
| *BAR_MON* | 12.11 | 5 | 2 | 0 | 0 | 0 |
|  | 12.25 | 4 | 2 | 0 | 0 | 0 |
| *SIC_MAZ* | 20.6 | 0 | 0 | 4 | 0 | 3 |
|  | 20.7 | 0 | 0 | 2 | 0 | 5 |
|  | 20.10 | 0 | 0 | 2 | 0 | 3 |
|  | 20.20 | 0 | 0 | 0 | 6 | 2 |

**Additonal file 2**. **Netrin haplotypes obtained in the cloning process for each individual**
