# Supplementary material for: Schmidtea mediterranea phylogeography: an old species surviving on a few Mediterranean islands?
Source: BMC Evol Biol. 2011 Sep 26;11:274. doi: 10.1186/1471-2148-11-274 (PMC3203090; doi:10.1186/1471-2148-11-274)
Supplement: Additional file 3 — Results of the SAMOVA analysis. A, W vs. C and S populations; B, W and C vs. S populations; C, W vs. C vs. S populations; D, W vs. C vs. TUN_LEB vs. Sicilian populations; E, W vs. S vs. SAR_SIL vs. C populations, except SAR_SIL. The percentage of variation among groups greatly increased in COI and CYB from 2 to 3 groups but remains almost unchanged from 3 to 4. Additionally, the percentage of variation among populations within groups greatly decreased in the three genes from 2 to 3 but decreased very little from 3 to 4. [file 1471-2148-11-274-S3.DOC]

|  |  | ***COI*** | | | ***CYB*** | | | ***N13*** | | |
| --- | --- | --- | --- | --- | --- | --- | --- | --- | --- | --- |
|  | **Numer of regionals groups** | 2 | 3 | 4 | 2 | 3 | 4 | 2 | 3 | 4 |
|  | **Scenarios** | **A** | **C** | **D** | **A** | **C** | **D** | **B** | **C** | **E** |
| **% variation** | **Among groups** | 61.8 | 98.08 | 98.82 | 74.41 | 92.57 | 95.3 | 51.4 | 55.22 | 56.65 |
|  | **Among populations within groups** | 37.89 | 1.59 | 0.83 | 23.9 | 5.45 | 2.61 | 20.75 | 12.08 | 10.02 |
|  | **Within populations** | 0.3 | 0.33 | 0.35 | 1.69 | 1.98 | 2.08 | 27.85 | 32.7 | 33.33 |

**Additional file 3. Results of the SAMOVA analyses. A**, *West* populations vs. *Central* and *Southeast*; **B**, *West* and *Central* vs. *Southeast*; **C**, *West* vs. *Central* vs. *Southeast*; **D**, *West* vs. *Central* vs. *TUN_LEB* vs. Sicilian populations; **E**, *West* vs. *Southeast* vs. *SAR_SIL* vs. *Central* populations except *SAR_SIL*. The % of variation among groups greatly increased in *COI* and *CYB* from 2 to 3 groups, but remain almost unchanged from 3 to 4, also the % of variation among populations within groups greatly decreased in the three genes from 2 to 3 but decreased very little from 3 to 4.
