# Supplementary figures and images for: Schmidtea mediterranea phylogeography: an old species surviving on a few Mediterranean islands?
Source: BMC Evol Biol. 2011 Sep 26;11:274. doi: 10.1186/1471-2148-11-274 (PMC3203090; doi:10.1186/1471-2148-11-274)

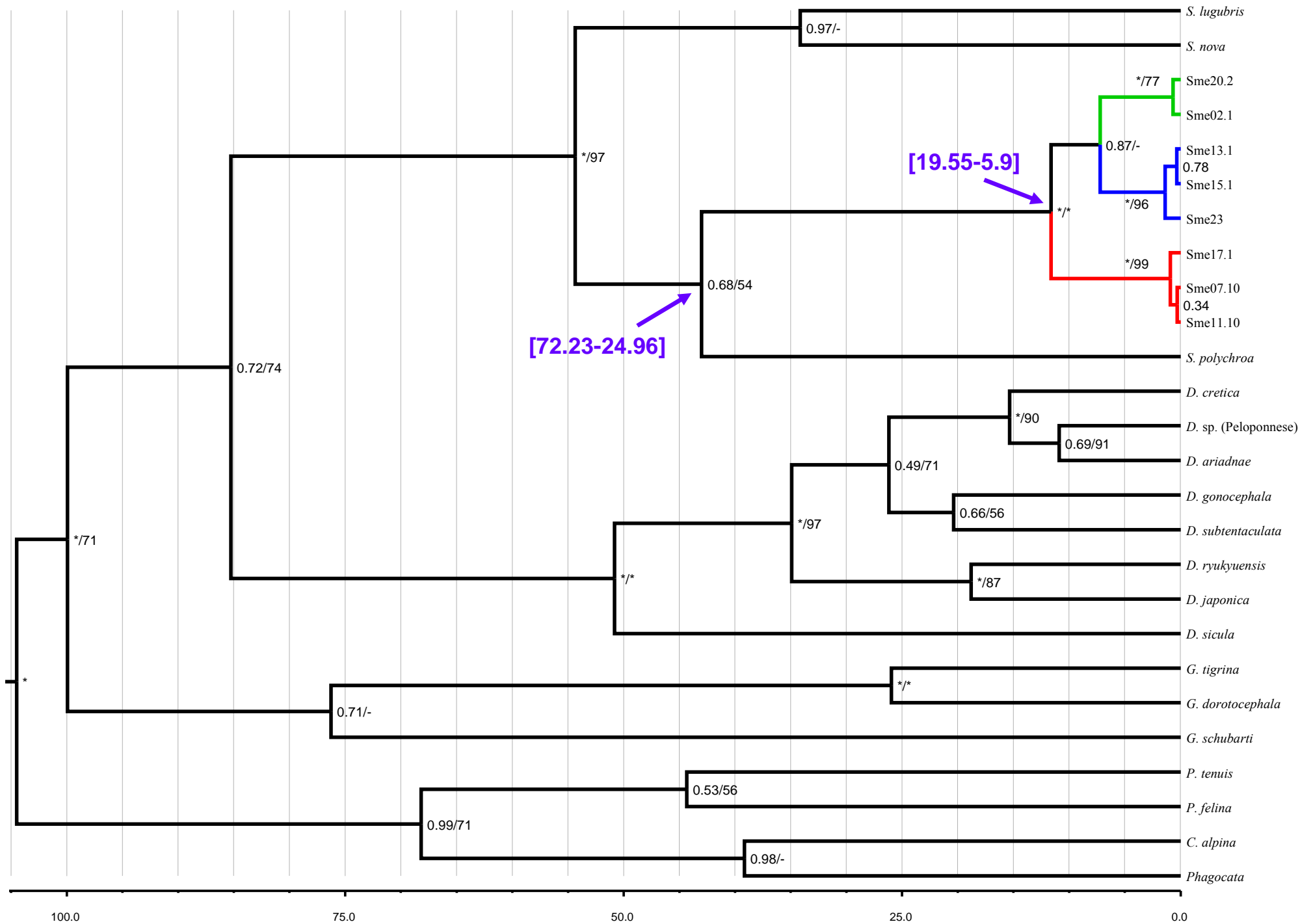

Supplement: Additional file 4 — Ultrametric tree obtained with BEAST to find the age of the split between S. mediterranea and S. polychroa. Numbers in nodes represent the posterior probability/bootstrap values obtained in the phylogenetic analyses performed with MrBayes and PHYML (not shown). * indicates the maximum value, and the symbol - indicates values <0.5 or 50%. Purple numbers represent the confidence interval (95%) for the age of the node. Coloured branches indicate the membership of S. mediterranea individuals to one of the geographical groups: W in red, C in blue and S in green. [file 1471-2148-11-274-S4.PDF]
