# Supplementary material for: Schmidtea mediterranea phylogeography: an old species surviving on a few Mediterranean islands?
Source: BMC Evol Biol. 2011 Sep 26;11:274. doi: 10.1186/1471-2148-11-274 (PMC3203090; doi:10.1186/1471-2148-11-274)
Supplement: Additional file 5 — The effect of asexual individuals on the dating analyses. [file 1471-2148-11-274-S5.PDF]

The effect of asexual individuals on the dating analyses

To perform this test, we hypothesised that when the fissiparous descendants of the sexual lineage overrun them, the asexual individuals essentially stop accumulating changes, and as a consequence, we expect the rate of substitutions in the branch that leads to the present day asexual populations to suddenly decrease. We attempted to model this behaviour by using BEAST. We considered the sequences from the asexual individuals to be fossils in the analyses, and by giving them different ages, we observed how that affects the ages for the splitting among the three groups. This strategy allowed us to approximate how the asexual lineages might be perturbing the dating analysis and, if this is the case, also to obtain a very rough estimate of their age.

We performed 5 tests by providing different times at which the asexual lineages could have appeared (23, 20, 15, 10 and 5 mya; in the results shown, the first tree was obtained in our second dating analysis), using a strict clock and providing a rate of 0.0027 for COI (which is not an estimate; this value was obtained for a broad group of planarians in our first dating analysis), without any calibration point.

Results: The basal splitting of the tree is always situated very close to the appearance of the asexual individuals (approximately 1 my before), which seems to indicate that the program finds the asexual sequences to be quite close to the ancestral sequence. The ages for the C vs. S splitting and for the basal splitting are congruent with the geological events when the asexual individuals are given an age of 20 or 23 my.

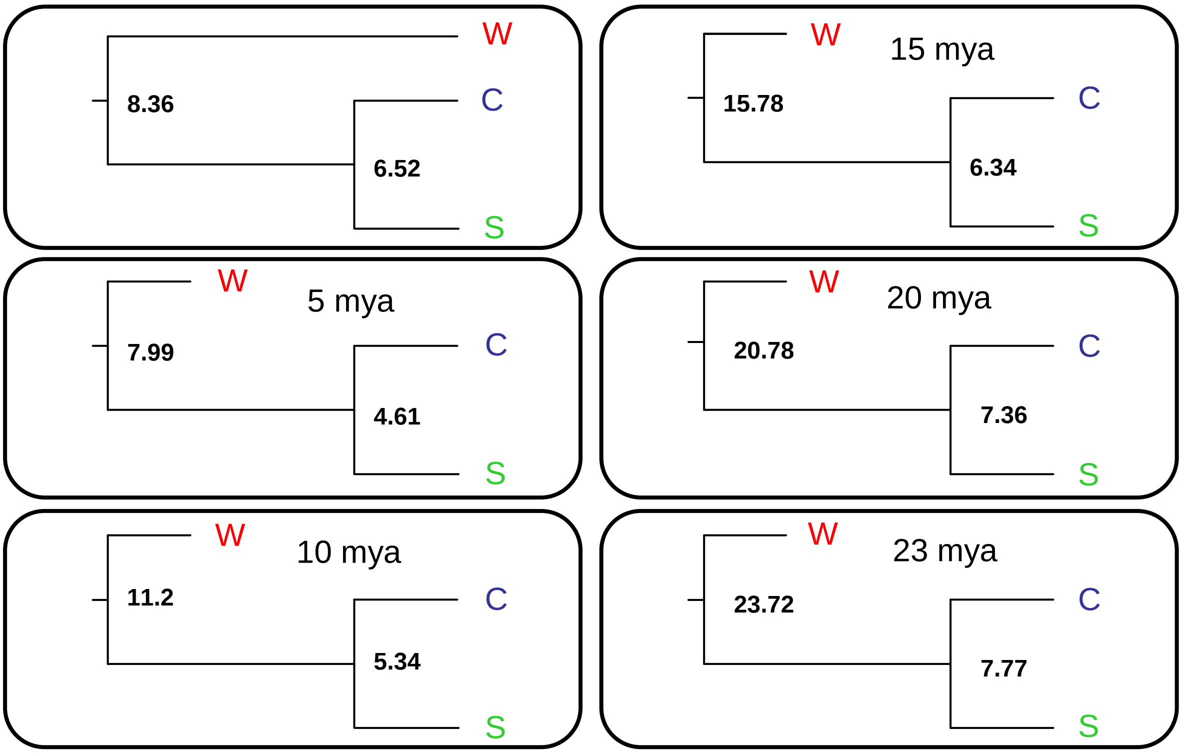

The analyses indicate that the asexual individuals will effectively perturb the inference of the ages in the phylogenetic tree if, as hypothesised, they do have an extremely low substitution rate owing to their method of reproduction. If this is the case, although considering them to be fossils may be too extreme, we cannot take the ages obtained here at face value; however, it can be envisaged that asexual individuals are probably quite old, probably having become fissiparous “soon” after the W group became independent.
